# Silencing of Rac1 and Arf6 reduces time-dependent and carbachol-induced contractions, proliferation, survival and growth in human bladder smooth muscle cells

**DOI:** 10.1007/s00345-025-05652-y

**Published:** 2025-05-07

**Authors:** Siwei Qian, Sheng Hu, Wenbin Zhu, Alexander Tamalunas, Christian G. Stief, Martin Hennenberg

**Affiliations:** 1 Department of Urology, LMU University Hospital, LMU Munich, Munich, Germany; 2 Urologische Klinik und Poliklinik, Marchioninistr. 15, 81377 München, Germany

**Keywords:** Overactive bladder (OAB), Lower urinary tract symptoms (LUTS), Storage symptoms, Bladder smooth muscle contraction, Rac1, Arf6

## Abstract

**Purpose:**

Storage symptoms in overactive bladder are explained by detrusor contractions and bladder wall thickening. Arf6 and Rac1 are monomeric GTPases with newly emerging roles in smooth muscle contraction and proliferation. Here, we investigated human bladder smooth muscle cells (hBSMC) functions after silencing of Arf6 or Rac1.

**Methods:**

hBSMC were transfected with Arf6- or Rac1-specific or scrambled siRNA (controls), and characterized using collagen contraction assays, for proliferation (EdU, Ki67), viability, growth in colony formation assays and actin organization.

**Results:**

Arf6 and Rac1 silencing was confirmed by RT-PCR. Time-dependent contractions (0.5–6 h after assay initiation) were reduced in Arf6- and Rac1-silenced cells (36% Arf6, 28% Rac1, after 6 h), compared to scramble-transfected cells. Carbachol (3 µM) increased the time-dependent contractions, which were reduced by silencing of Arf6 (40–62%, 0.5–6 h) or Rac1 (30–59% at 0.5–6 h). With U46619 or endothelin-1, time-dependent contractions were similar to contractions without agonists, but again reduced in Arf6- and Rac1-silenced cells. Compared to scramble-transfected cells, silencing reduced the proliferation rate (Arf6 52%, Rac1 33%), Ki67 mRNA expression (89%, 91%), colony formation (63%, 66%), viability (Arf6 up to 84%, Rac1 up to 85%), and actin polymerization (30%, 31%).

**Conclusions:**

Arf6 and Rac1 promote time-dependent and carbachol-induced contractions of hBSMC, which may be mediated by actin polymerization. Simultaneously, Arf6 and Rac1 promote proliferation, growth and survival in hBSMC. Arf6 and Rac1 may be potentially involved in detrusor overactivity, bladder wall thickening and medical treatment of overactive bladder.

**Supplementary Information:**

The online version contains supplementary material available at 10.1007/s00345-025-05652-y.

## Introduction

Bladder smooth muscle contraction by muscarinic receptors induces voiding, while uncontrolled detrusor contractions and bladder wall thickening may cause storage symptoms in overactive bladder (OAB) [1]. Intracellular signal transduction in bladder smooth muscle contraction involves the monomeric GTPase RhoA, which mediates contraction by activation of Rho kinase, and promotes proliferation in all smooth muscle-rich organs [2, 3]. In bladder smooth muscle, RhoA is activated by carbachol [4] and upregulated by mechanical stretch [5], while small molecule Rho kinase inhibitors inhibit contractions of isolated detrusor tissues and improve cystometric parameters in OAB models [1]. Analog roles are now emerging for other GTPases, including Rac1, Arf6 and different smooth muscle types [3].

Arf6-mediated contraction was first described in a knockout model for the prostate [6], but similar evidence for the bladder is lacking. Rac1-mediated detrusor contraction has been suggested for rodents, and indirectly in human cells and tissues using small molecule inhibitors with limited specificity [3, 7]. Carbachol-induced bladder smooth muscle contractions were found to be inhibited by smooth muscle-specific Rac1 knockout in mice, and by Rac inhibitors in human, mouse and rat bladder tissues [7–9]. However, evidence from silencing or knockout models for validation in human bladder cells, or for non-cholinergic contractions is still lacking. In parallel, Rac1 and Arf6 have been suggested to promote proliferation [6, 10]. Rac1 expression was found to be upregulated in bladders of rats and mice with streptozotocin-induced diabetes, which was associated with enhanced cholinergic contractility, oxidative stress, and increases in bladder wall thickness [8, 11]. In addition, Rac1 expression in human bladder smooth muscle cells is upregulated by hydrostatic pressure, leading to enhanced proliferation [12]. Accordingly, a role of Rac1 has been proposed in OAB and other bladder pathologies [13], and similar roles appear possible for Arf6. However, evidence directly linking Rac1 or Arf6 to contractility and growth in human bladder smooth muscle cells through silencing or knockout approaches is lacking, and definitive associations with clinical conditions in human samples have yet to be established. Here, we addressed the role of Rac1 and Arf6 for contraction and growth-related functions of human bladder smooth muscle cells.

## Methods

### Cell culture

Human bladder smooth muscle cells (hBSMC) were obtained from Sciencell Research Laboratories (Carlsbad, CA, USA) (catalog #4310, lot #5828), and cultured as described in supplementary materials. Information about the sex, gender or other factors (age, presence of disease, background of sample, and others) of donors was not provided and was not available upon request.

### Silencing of Rac1 and Arf6

hBSMC were transfected with scrambled siRNA (Silencer^®^ Select scrambled Negative Control siRNA duplex, 4390843), human Rac1 siRNA (Silencer Select predesigned siRNA, s11712, sequence CTGTTTCTCTGCAGTTTTCtt), or human Arf6 siRNA (s1565, sequence AGACGGUGACUUACAAAAAtt) (Ambion Silencer Select library, Life Technologies, Carlsbad, CA, USA). siRNAs were diluted in Opti-MEM to final concentrations of 50 nM, and transfection was performed as described in supplementary materials using the Stromal Cell (PrSC) Avalanche™ Transfection Reagent (EZ Biosystems, College Park, MD, USA). Following transfection, cells were cultured for 3 d, until RT-PCR or assays were performed, with the following exceptions. For contraction assays, cells were placed into collagen plugs and contractions were assessed after 0.5–6 h. For viability assays, cells were cultured for 1–3 d after transfection. Non-transfected cells (“wildtype”) were cultured under identical conditions. For colony formation assays, cells were placed onto plates and cultured for 13 days.

### RT-PCR

Isolation of mRNA and real-time PCR (RT-PCR) were performed as described in supplementary materials. Ready-to-use primers were purchased from Qiagen (Hilden, Germany), based on the RefSeq accession numbers NM_006908 for Rac1 (PPH00733F-200), NM_001663 for Arf6 (PPH10416A-200), NM_002417 for Ki-67 (PPH01024E-200) and NM_002046 for GAPDH (PPH00150F-200). Results were calculated as 2^−∆Ct^ and normalized to means of controls.

### Matrix contraction assays

Contractions were assessed using the CytoSelect 24-Well Cell Contraction Assay Kit (Cell Biolabs, San Diego, CA, USA), as described in supplementary materials. Pictures were taken 0.5–6 h after addition of medium. Results are expressed as contraction of the matrix plug in mm.

### EdU assays

Proliferation rates were assessed using a 5-ethynyl-2′-deoxyuridine (EdU)-based assay kit (catalog number BCK-EdU555IM100, Baseclick, Tutzing, Germany) as described in supplementary materials, after seeding of 10,000 cells/well into coverslides, incubation, and transfection after a further 24 h at a confluence of 70%. The proliferation rate was expressed as the percentage of EdU-stained cells from all cells within a microscopic field.

### CCK-8 assays

Viability was assessed using the Cell Counting Kit-8 (CCK-8) (Sigma Aldrich, Munich, Germany), as described in supplementary materials. 5,000 cells were seeded per well of 96-well plates. Cells were cultured for further 24 h before transfection. Finally, assays were performed after 24–72 h, and optical densities (OD) at 450 nm in all wells were determined using a microplate reader. Results are reported as ODs in diagrams, and as percentage descrases (from means for controls) in the text.

### Colony formation assays

One hundred transfected cells were placed per well of 6-well plates and cultured for 13 days, before colonies were stained as described in supplementary materials. Visible colonies were counted from pictures, and results are reported as number of colonies (n) per well.

### Phalloidin stainings

Cells were plated into 12-well chambered coverslides (10,000 cells/well), and transfected at a confluence of 70% after 24 h. After 72 h, cells were stained with fluoresceine isothiocyanate- (FITC-) labelled phalloidin (Sigma-Aldrich, Munich, Germany) and analyzed as described in supplementary materials.

### Statistical analyses

Data were analyzed using Graphpad Prism (GraphPad Software Inc., San Diego, CA, United States). Comparisons in data sets containing two groups were performed by a two-tailed, unpaired t-test. Groups in contraction assays were compared by two-way ANOVA, without multiple comparison. Data containing three groups were analyzed by one-way ANOVA followed by Tukey’s multiple comparison for comparison of all three groups with each other, or by one-way ANOVA followed by Dunnett’s multiple for comparison of silenced cells to the shared scramble group. P values < 0.05 were considered significant. Our study shows an exploratory character, so that p values need to be considered as descriptive. Effect sizes were reported as folds or percent decreases of means of controls, with 95% confidence intervals (CI).

### Drugs and nomenclature

Carbachol is a muscarinic receptor agonist and was dissolved in deionized water before application [14]. U46619 is a thromboxane A_2_ receptor agonist and was dissolved in ethanol [14]. Endothelin-1 was dissolved in deionized water. Tolterodine is a subtype-unselective muscarinic antagonist and is available for treatment of storage symptoms [1, 14], and was dissolved in dimethylsulfoxide (DMSO). Carbachol was purchased from Sigma (Munich, Germany), U46619 and endothelin-1 were obtained from Enzo Life Sciences (Lörrach, Germany), and tolterodine tartrate from Tocris (Bristol, UK).

## Results

### Silencing of Rac1 and ARF6

**Fig. 1:**
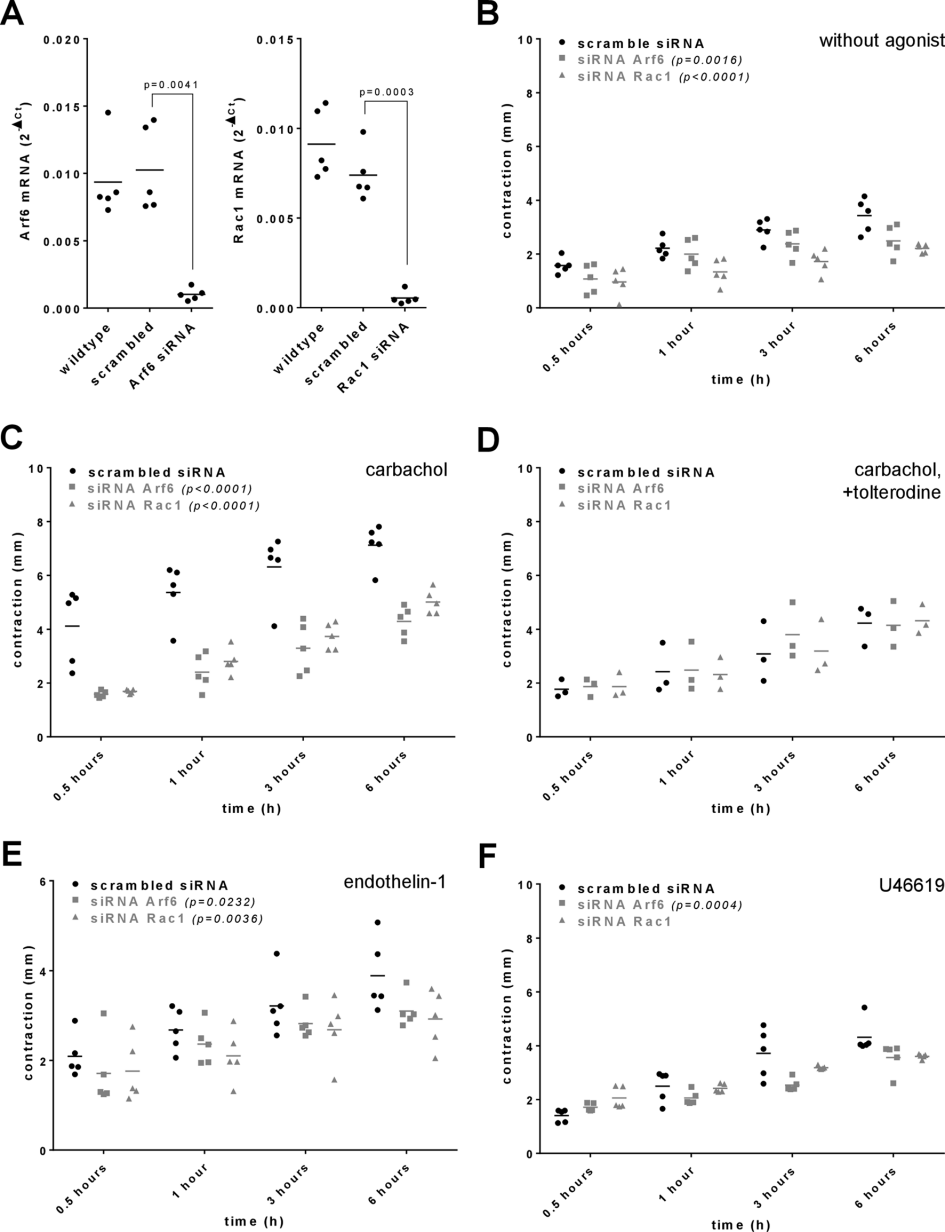
Silencing of Arf6 and Rac1, and contractions in hBSMC. **(A)** Expression of Arf6 or Rac1 mRNA was assessed by RT-PCR, 72 h after transfection with Arf6- or Rac1-specific siRNA, or with scramble siRNA, and in non-transfected cells (“wildtype”). Shown are all values from a total of five independent experiments, together with means and p values from one-way ANOVA followed by Tukey’s multiple comparisons test. **(B-F)** Time-dependent contractions (0.5–6 h) were assessed by collagen-matrix contraction assays, without agonists (**B**), in the presence of carbachol (300 nM) (**C**), in the presence of carbachol (3 µM) and tolterodine (1 µM) (**D**), in the presence of endothelin-1 (10 µM) (**E**) or in the presence of U46619 (300 nM) (**F**). Shown are all values and means from a total of five independent experiments per diagram, with the exception of tolterodine experiments (**D**), which are based on a total of three experiments, together with p values from two-way ANOVA for comparison of siRNA groups to corresponding scramble groups (in upper left parts of diagrams). P values ≥ 0.05 are not indicated, so that so that lacking values reflect no significance

Transfection with Rac1 or Arf6 siRNA for 72 h downregulated Rac1 mRNA expression, compared to scramble controls and wildtype cells (Fig. 1a). Rac1 mRNA amounted to 0.07 fold [0.01–0.13], and Arf6 mRNA levels to 0.1 fold [0.04–0.16] of levels in scramble controls. Expressions were similar between scramble controls and wildtype hBSMC (Fig. 1a).

### Silencing of Rac1 and Arf6 reduces time-dependent contractions

Following assay initiation by serum addition, cells showed time-dependent contractions (Figs. 1b-f). Contractions were decreased in Arf6- and Rac1-silenced cells, compared to scramble controls. Six hours after assay initiation, contractions were reduced by 28% [7–48] in Arf6-silenced cells, compared to scramble controls after six hours (Fig. 1b). Contractions in Rac1-silenced cells were reduced by 39% [-2 to 80] after 0.5 h, 40% [13–66] after 1 h, 41% [22–59] after 3 h, and 36% [30–42] after 6 h, compared to scramble controls at the corresponding time points (Fig. 1b).

### Silencing of Rac1 and Arf6 reduces carbachol-induced contractions

With carbachol (3 µM), contractions were again decreased in Arf6- and Rac1-silenced cells, compared to scramble controls (Fig. 1c). In Arf6-silenced cells, contractions were decreased by 62% [58–65] after 0.5 h, 55% [40–70] after 1 h, 48% [29–66] after 3 h, and 40% [30–50] after 6 h, compared to scramble controls with carbachol (Fig. 1c). In Rac1-silenced cells, contractions were decreased by 59% [57–61] after 0.5 h, 48% [37–59] after 1 h, 41% [31–51] after 3 h, and 30% [22–38] after 6 h, compared to scramble controls with carbachol (Fig. 1c).

In the presence of tolterodine (1 µM) and carbachol (3 µM), contractions were similar between cells transfected with Ar6-specific, Rac1-specific and scramble siRNA (Fig. 1d). Overall, contractions in the presence of tolterodine in all three groups amounted to ranges of contractions in Rac1- and Arf6-silenced cells with carbachol (Figs. 1c, d). Thus, tolterodine fully inhibited carbachol-induced contractions (Figs. 1c, d).

### Silencing of Rac1 and Arf6 reduces contractions with endothelin-1- and U46619

With endothelin-1 (10 µM), contractions were partly reduced by silencing of Arf6 and Rac1, compared to scramble controls (Fig. 1e). In Arf6-silenced cells, contractions were decreased by 18% [-28 to 65] after 0.5 h, 12% [-10 to 33] after 1 h, 12% [-1 to 26] after 3 h, and 20% [8–32] after 6 h, compared to scramble controls with endothelin-1 (Fig. 1e). In Rac1-silenced cells, contractions were decreased by 16% [-25 to 57] after 0.5 h, 21% [-5 to 48] after 1 h, 17% [-10 to 43] after 3 h, and 25% [4–45] after 6 h, compared to scramble controls with endothelin-1 (Fig. 1e).

With U46619 (300 nM), contractions were reduced partly by silencing of Arf6 and Rac1 in later assay phases, compared to scramble controls (Fig. 1f). In Arf6-silenced cells, contractions were decreased by 18% [5–30] after 1 h, 32% [24–40] after 3 h, and 17% [2–33] after 6 h, compared to scramble controls with U46619 (Fig. 1f). In Rac1-silenced cells, contractions were reduced by 14% [12–16] after 3 h, and 16% [14–19] after 6 h, compared to scramble controls with U46619 (Fig. 1f).

### Silencing of Rac1 and Arf6 decreases proliferation rate and Ki-67 mRNA expression

**Fig. 2:**
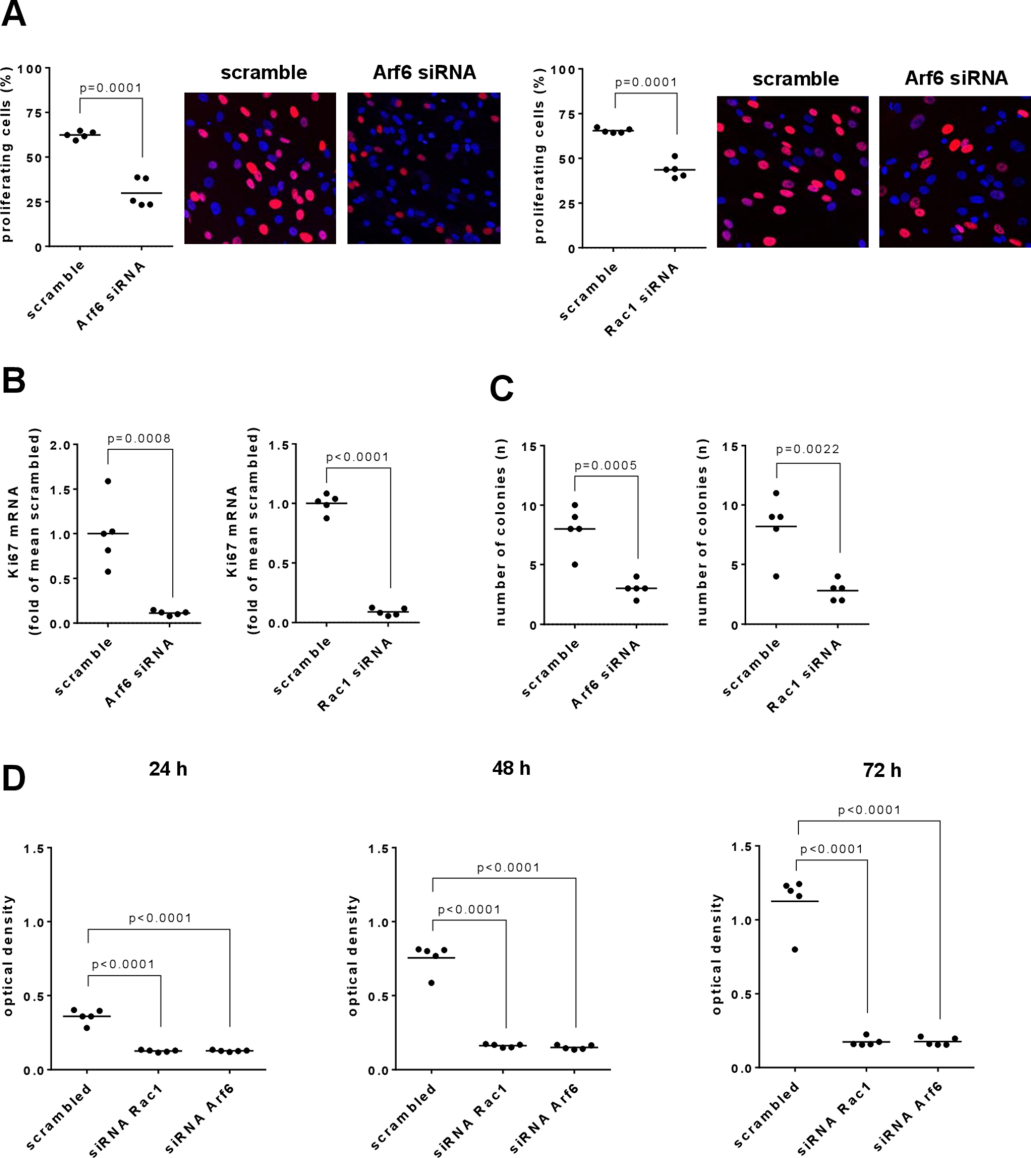
Proliferation rate, Ki67 expression, viability and colony formation in hBSMC. **(A)** Proliferation rates were determined by EdU assays, 72 h after transfection with Arf6- or Rac1-specific siRNA, or with scramble siRNA. Red, proliferating cells; blue, non-proliferating cells. **(B)** Expression of Ki67 mRNA was assessed by RT-PCR, 72 h after transfection with Arf6- or Rac1-specific siRNA, or with scramble siRNA. **(C)** Colony formation over 13 days was determined after transfection with Arf6- or Rac1-specific siRNA, or with scramble siRNA. **(D)** Viabilities were determined by CCK-8 assays, 24 h, 48–72 h after transfection with Arf6- or Rac1-specific siRNA, or with scramble siRNA. Shown are all values and means from five experiments per diagram, together with p values from two-tailed t-test in (**A**) to (**C**), and from one-way ANOVA followed by Dunnett’s multiple comparison test in (**D**)

Transfection with Rac1 siRNA reduced the proliferation rate in EdU assays by 33% [24–42], and transfection with Arf6 siRNA by 52% [37–68] (Fig. 2a). The percentage of proliferating cells amounted to 65% [64–67] in scramble controls and to 44% [38–50] in Rac1 siRNA-transfected cells, and to 62% [60–65] in scramble controls for Arf6 and to 30% [20–40] in Arf6 siRNA-transfected cells (Fig. 2a). In parallel, transfection with Rac1 or Arf6 siRNA downregulated Ki-67 mRNA (Fig. 2b). Ki-67 mRNA amounted to 0.09 fold [0.05–0.13] in Rac1 siRNA-transfected hBSMC, and to 0.11 fold [0.08–0.15] in Arf6 siRNA-transfected hBSMC, compared to corresponding scramble controls.

### Silencing of Rac1 and Arf6 inhibits colony formation

Transfection with Rac1 siRNA reduced the number of colonies by 66% [53–79], from 8.2 colonies/well [5-11.4] with scramble siRNA to 2.8 colonies/well [1.8–3.8] with Rac1 siRNA (Fig. 2c). Transfection with Arf6 siRNA reduced the number of colonies by 63% [52–73], from 8.0 colonies/well [5.7–10.3] with scramble siRNA to 3.0 colonies/well [2.1–3.9] with Arf6 siRNA (Fig. 2c).

### Silencing of Rac1 and Arf6 reduces viability

Transfection with Rac1 siRNA reduced the viability by 65% [63–68] after 24 h, 79% [77–80] after 48 h, and 85% [81–88] compared to scramble siRNA-transfected controls. Similarly, transfection with Arf6 siRNA reduced the viability by 65% [63–67] after 24 h, 80% [78–82] after 48 h, and 84% [82–87] compared to scramble siRNA-transfected controls.

### Silencing of Rac1 and Arf6 impairs actin organization

**Fig. 3:**
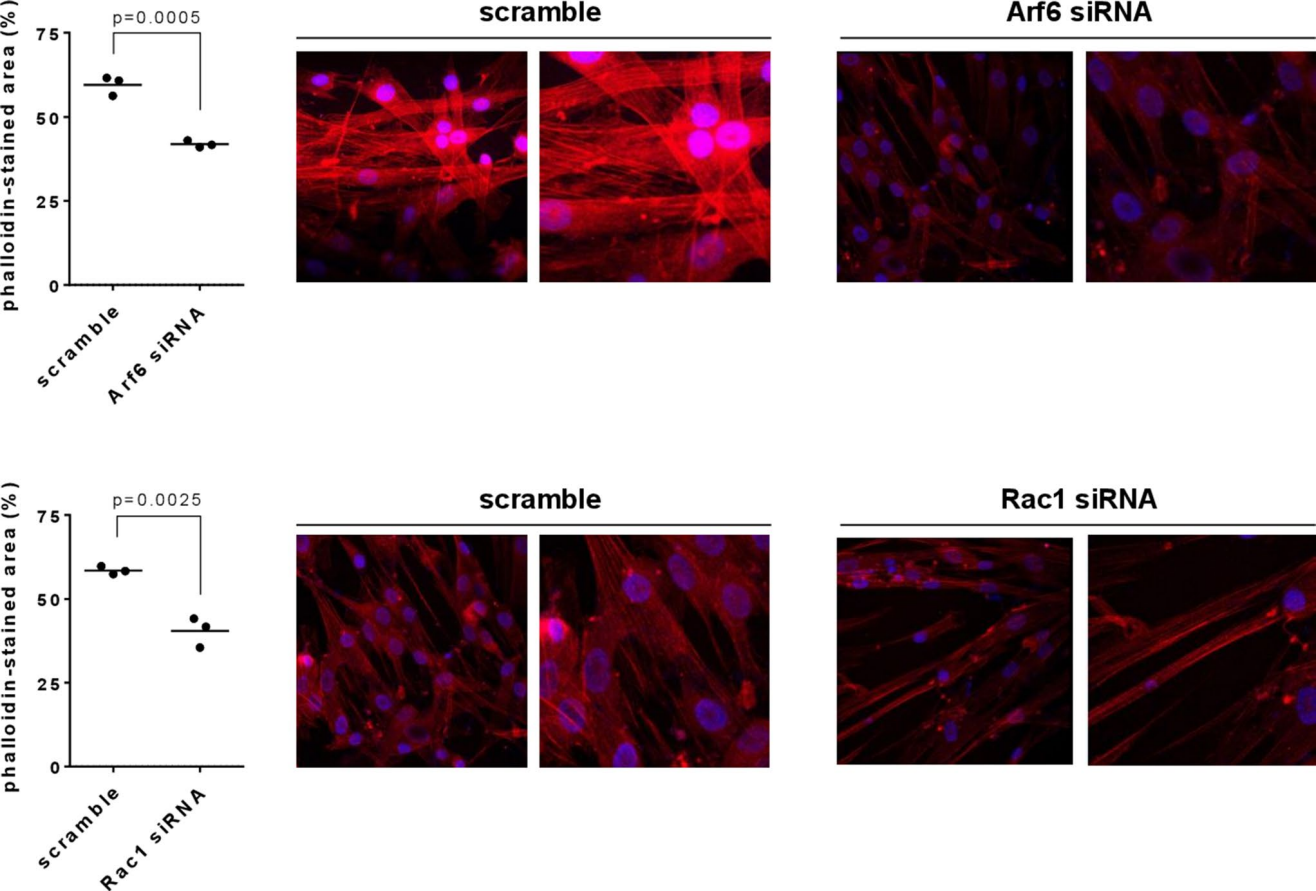
Actin organization in hBSMC. Polymerized actin was stained by phalloidin, 72 h after transfection with Arf6- or Rac1-specific siRNA, or with scramble siRNA. Shown are all values and means from three experiments per diagram, together with p values (descriptive, because based on three experiments) from two-tailed t-test and with representative pictures (original, and cutouts from the corresponding original image)

In scramble siRNA-transfected cells, actin was organized to filaments, forming bundles and long fibers. Transfection with Rac1 or Arf6 siRNA reduced the number of actin filaments and the intensity of fluorescence (Fig. 3). However, remaining actin was still organized to filaments (Fig. 3). Actin-stained areas were reduced by 31% [12–50] by transfection with Rac1 siRNA and by 30% [25–34] by transfection with Arf6 siRNA, compared to scramble controls. Actin-stained areas amounted to 58% [56–61] in scramble controls and to 40% [29–51] in Rac1 siRNA-transfected cells, and to 60% [52–67] in scramble controls and to 42% [39–44] in Arf6 siRNA-transfected cells.

## Discussion

Detrusor contractions mediate bladder emptying in normal voiding, and are central in pathophysiology and treatment of OAB [1]. In addition, OAB can be accompanied by bladder wall thickening [1]. Our findings suggest that contraction and proliferation of bladder smooth muscle cells are promoted by both Rac1 and Arf6. Following extensive research confirming contributions of RhoA in contraction and proliferation in all smooth muscle-rich organs, analog roles of other GTPases are now emerging, including Rac1 and Arf6 [3]. Procontractile roles of Rac1 have been documented for several smooth muscle types, including bladders of Rac1 knockout mice [9]. Evidence for a procontractile role of Arf6 is available from knockout in prostate smooth muscle cells [6], but from no other organ. Thus, our data are the first supporting Rac1- or Arf6-mediated contraction in human bladder smooth muscle, or Arf6-mediated proliferation and growth of bladder smooth muscle cells.

Contractions in collagen matrix assays were enhanced by carbachol. This effect was sensitive to the muscarinic antagonist tolterodine, which is clinically used for medical treatment of storage symptoms in OAB [1, 15, 16]. Both silencings reduced contractions without agonists, and in the presence of carbachol. Without agonists, reductions in time-dependent contractions occurred over the complete assay period in Rac1-silenced cells, and were most obvious at the end of the assay in Arf6-silenced cells, possibly reflecting divergent time-dependent kinetics in the contributions of Rac1 and Arf6 to contractions. Reductions of cholinergic contractions were similar with both silencings, and of similar degree to effects of tolterodine. Thus, our findings may suggest that Rac1 and Arf6 are activated in muscarinic receptor-mediated contractions, similarly to the well-documented activation of RhoA by contractile smooth muscle receptors [2, 17, 18, 19].

Endothelin-1 and U46619 did not effectively enhance time-dependent contractions in our assays with hBSMC, but previously induced contractions in human bladder tissues using the same concentrations [20–22]. Notably, however, contractions with endothelin-1 and U46619 were again decreased in Rac1- and Arf6-silenced cells. The physiologic roles of endothelin- and thromboxane-induced bladder contractions are unknown. Probably, they are not involved in voiding contractions, but microcontractions in OAB are of supposed non-cholinergic origin [1]. However, whether microcontractions in OAB, or microcontractions initiating the voiding reflex involve endothelin-1 or thromboxane, remains uncertain. Given the importance of detrusor contractility in health and disease, and the contractile forces observed with these agonists in human detrusor tissues [20–22], this gap in understanding the functional relevance of endothelin-1- and thromboxane-induced detrusor contractions is noteworthy. Compared to cholinergic and purinergic contractions, these contractions have remained largely unexplored, what prompted us to include these agonists in our study. It is tempting to assume that the contractile responses to endothelin-1 and thromboxane in the detrusor must have a defined physiological or pathophysiological role, e.g. by fine-tuning of bladder smooth muscle tone or in the voiding reflex. However, it remains possible that these strong non-cholinergic responses serve no essential physiological function, as not each biological and evolutionary feature is adaptive.

Time-dependent contractions in our assays occurred without exogenous agonists, but required addition of serum. Serum-induced contractions may in part be mediated by endothelin-1 or thromboxane A_2_, which are known to be present in standard serum preparations. This may explain the lack of additive responses to exogenous endothelin-1 and U46619, as receptor activation might already be saturated by agonists from the serum. Other serum components, including thrombin or transforming growth factor-β, may contribute as well to time-dependent contractions of smooth mucle cells and fibroblasts [23, 24]. Our assay reflects contractions over hours and requires an initial matrix polymerization, so that rapid contractions within minutes are excluded. Thus, kinetics in our assay are more compatible with tonic than phasic contractions. This may limit conclusions regarding voiding contractions, which occur within seconds or minutes. On the other hand, tonic contractions in isolated detrusor tissues have been commonly used for precontraction in relaxation experiments with clinically used drugs, including β_3_-adrenergic agonists.

Our findings may suggest Rac1- and Arf6-driven growth of hBSMC, and possibly functions in bladder wall thickening. Apart from proliferation, changes in apoptosis and cell death may contribute to reduced viability and colony formation, so that this may be the subject of a follow-up study. Bladder wall thickening by detrusor hypertrophy may contribute to storage symptoms, but its precise role is less understood than detrusor overactivity [1]. OAB includes a wide range of clinical conditions, from neurogenic bladder dysfunction, through detrusor overactivity (with probably different forms) to storage symptoms and incontinence even with an acontractile detrusor [1, 16]. Obviously, contraction and growth in the detrusor are no separate functions, but may be partly connected by Rac1- and Arf6-mediated mechanisms.

The simultaneous impacts of both silencings on contractility, proliferation, and overall survival and growth suggest that these functions are co-regulated by Rac1 and Arf6. While the identification of mechanisms and molecular partners in this Rac1- and Arf6-mediated regulation were beyond the scope of this study, potential contributors may provisionally include phenotypic changes, or any other mechanism affecting actin organization and actin-dependent functions. Notably, both contractility and proliferation are tightly dependent on intact actin dynamics. A classical phenotypic de-differentiation, known from smooth muscle cells and including loss of contractility with simultaneous gains in proliferative (and synthetic) activity [25] does obviously not account for the phenotype in Rac1- and Arf6-silenced cells. Although we can not exclude partial phenotypic alterations, the pattern observed in our silenced cells is inconsistent with the typical phenotypic switch from contractile to synthetic smooth muscle cells [25], which is usually characterized by increased proliferation. Future studies should address this in more detail, by assessment of phenotypic and other markers.

Similar to our findings, isolated detrusor tissues from Rac1 knockout mice showed impaired cholinergic contractions [9]. Data confirming an analog role in the human bladder by silencing or knockout were not yet available, but the Rac inhibitors NSC27366 and EHT1864 previously inhibited neurogenic and carbachol-induced contractions of human detrusor tissues [7], and cholinergic contractions in rat bladder tissues [8]. However, their specificity is limited [7, 26]. In addition to the bladder, Rac1-mediated smooth muscle contraction has been suggested for other organs as well [3]. Evidence for a procontractile role of Arf6 was so far limited to prostate smooth muscle cells [6]. Acknowledging that silencing of Rac1 and Arf6 reduced hBSMC contractions, it could be claimed that these GTPases are candidate targets for future OAB treatment, in experimental models or trials. In fact, small molecule inhibitors exist for both GTPases [3, 6, 7, 26], but their tolerability needs to be examined and may limit a clinical application.

## Conclusions

Rac1 and Arf6 promote time-dependent and carbachol-induced contractions, proliferation and growth in hBSMC. Rac1 and Arf6 are novel pathways of contraction and growth in hBSMC, and may be involved in detrusor overactivity, bladder wall thickening and/or OAB.

## Electronic supplementary material

Below is the link to the electronic supplementary material.

Supplementary Material 1

## Data Availability

The authors declare that all the data supporting the findings of this study are contained within the paper. Original and raw data containing all individual data points are available as supplemental information.
