# Supplementary material for: Silencing of Rac1 and Arf6 reduces time-dependent and carbachol-induced contractions, proliferation, survival and growth in human bladder smooth muscle cells
Source: World J Urol. 2025 May 7;43(1):279. doi: 10.1007/s00345-025-05652-y (PMC12058880; doi:10.1007/s00345-025-05652-y)
Supplement: Supplementary file 1 — Supplementary Material 1 [file 345_2025_5652_MOESM1_ESM.pdf]

Silencing of Rac1 and Arf6 reduces time-dependent and carbachol-induced contractions, proliferation, survival and growth in human bladder smooth muscle cells

World Journal of Urology

Siwei Qian, Sheng Hu, Wenbin Zhu, Alexander Tamalunas, Christian G. Stief, Martin Hennenberg

Department of Urology, LMU University Hospital, LMU Munich, Munich, Germany

Martin.Hennenberg@med.uni-muenchen.de

Methods: details and protocols

1 Cell Culture

1.1 Cell Line

Experiments were performed in human bladder smooth muscle cells (BSMCs) obtained from ScienCell Research Laboratories (Carlsbad, CA, USA), which are primary myocytes isolated from the urinary bladder of a human donor and can be commercially purchased (catalog number #4310). Cell culture was performed using a medium designed for smooth muscle cells (Smooth Muscle Cell Medium (SMCM), #1101, ScienCell), combined with 2% Fetal Bovine Serum (FBS) (#A5256801, ThermoFisher Scientific, Waltham, MA, USA), 1% human bladder smooth muscle cell growth supplement (#1152, ScienCell) and 1% penicillin/streptomycin (#10378-016, ThermoFisher). Cells from passages 2 to 5 were maintained under conventional culture conditions at 5% CO_2 and 37°C.

1.2 Cell Thawing

Cells were thawed using the following protocol:

1. After removal of the vial from the liquid nitrogen tank, cells in the vial were quickly thawed at 37°C in a water bath.
2. Pre-warmed smooth muscle cell medium mixed with 2% FBS, 1% human bladder smooth muscle cell growth supplement and 1% penicillin/streptomycin solution was applied to cultivate thawed cells, followed by seeding one million cells per T75 flask and maintaining the total volume of cell culture medium at 10 mL.
3. Flasks were maintained under 37°C in a 5% CO_2 environment in an incubator.

1.3 Cell passaging and cryopreservation

Cell passaging and cryopreservation were conducted according to the following protocol:

1. The medium in the flasks was aspirated, followed by washing with pre-warmed PBS twice.
2. 1-2 mL Trypsin-EDTA per flask was distributed to cover the cells completely.
3. After being put back to the incubator for 2 min and subsequently detachment from the flask surface, 10 mL of Smooth Muscle Cell Medium comprising penicillin/streptomycin solution were added per flask, followed by resuspension to stop the enzymatic activity.
4. The medium containing cell pellets was centrifuged at 1,300 rpm for 5 min to collect cell sediments, which were resuspended in pre-warmed cell medium and seeded in new flasks.
5. For cryo-preservation, re-suspended cells were combined with freezing medium to achieve the final concentration, composed of 10% DMSO, 40% FBS, and 50% cell suspension. Subsequently, cells were cryopreserved for short-term storage at -80°C or for long-term storage in liquid nitrogen.

2 siRNA Transfection

The Prostate stromal cell Avalanche^{TM} transfection reagent (catalog number #EZT-PRSC-1, EZ Biosystems, College Park, MD, USA) was utilized to transfect hBSMCs. Antibiotic-free medium was used for culturing cells. Upon 24 h before transfection, cells reached 70% confluence. RT-PCR and other experiments were conducted at least 72 h after transfection had been completed. CCK8 and EDU assays were carried out after an extra 24 h. Predesigned siRNAs (Silencer Select predesigned siRNA, Ambion Silencer Select library) for Rac1 (s11712) or Arf6 (S1565), and scrambled siRNA for negative controls (Silencer® Select scrbambled Negative control siRNA duplex, #4390843) were purchased from Life Technologies (Carlsbad, CA, USA). Transfection was carried out according to the following protocol:

1. The Rac1 siRNA (sequence: CTGTTTCTCTGCAGTTTTCtt), Arf6 siRNA (sequence: AGACGGUGACUUACAAAAAtt), and Silencer Select scramble siRNA (Silencer® Select scrambled Negative Control siRNA duplex, 4390843) were diluted in OptiMEM® (catalog number #31985-070, ThermoFischer), reaching concentrations of 50 nM, followed by vortexing.

2. Transfection reagent was combined with siRNA diluted in OptiMEM® at a final volume ratio of 1:40. The mixture was then vortexed for 15 sec. For all three types of siRNA, the same procedure as described above was followed.

3. All three kinds of transfection master-mixes were incubated at room temperature for 15 min.

4. 100 µL of transfection mix was added to every milliliter of medium without penicillin/streptomycin solution, i. e. pipetted into flask or plate.

5. Plates or flasks were carefully swirled, then they were centrifuged at 300 g for 5 min at room temperature.

6. Plates or flasks were placed in incubator for 5 h (37°C).

7. Pre-warmed (37°C) fresh medium with penicillin/streptomycin was added after removing the initial one. Transfected cells were cultured for an additional 72 hours for further analyses.

3 Real Time Polymerase Chain Reaction (RT-PCR)

3.1 RNA Extraction and Quantification

RNA extraction and quantification were carried out using the RNeasy Kit for RNA Purification (catalog number #74104 Qiagen, Hilden, Germany), according to the following protocol:

1. Cells were seeded in 6-well plates, with a density of approximately 1 x 10^6 cells in each well.
2. The wells were washed twice with phosphate-buffered saline (PBS) after removing the medium. Subsequently, 350 µL buffer RLT (provided with the kit) and 350 µL 70% ethanol were added together to each well and mixed thoroughly.
3. Homogenization of the mixed buffer. Subsequently, the lysate was transferred into QIA-shredder spin columns (provided with the kit) placed into 2 µL collection tubes (provided with the kit).
4. After centrifugation of 1 min at 10,000 g, the throughput was discarded.
5. 700 µL of RW1 (provided with the kit) was added to columns, and original collection tubes were replaced by new ones, followed by centrifugation at 10,000 g centrifugation for 15 seconds, with columns retained.
6. Step 5 was repeated.
7. 500 µL of RPE (provided with the kit) was added, followed by centrifugation at 10,000 g of centrifugation for 15 sec, with collection tube replaced.
8. The same volume of Buffer RPE was added again, followed by centrifugation for 1 min at 10,000 g.
9. All spin columns were transferred to 1.5 mL collection tubes. Subsequently, 30 µl of nuclease-free water (provided with the kit) was added directly onto membrane inside. The isolated RNA inside tubes was kept after centrifugation at 10,000 g lasting for 2 min.

10. Quantification of RNA was performed using a spectrophotometer (NanodropTM, ThermoFisher).

3.2 Reverse Transcription

Reverse transcription was carried out using the QuantiTect Reverse Transcription Kit (catalog number #205313, Qiagen), according to the following protocol:

1. 1 µg of total RNA per sample was diluted with nuclease-free water to a total volume 12 µL, followed by mixing with 2 µL gDNA wipeout buffer (provided with the kit). Thus, the total mixture volume was 14 µL. Tubes were then incubated at 42°C for 2 min.
2. Any bubbles were removed by brief centrifugation. Subsequently, tubes were immediately placed on ice.
3. 1 µL of Quantiscript Reverse Transcriptase (provided with the kit), 4 µL of Quantiscript RT Buffer (provided with the kit), 1 µL of RT Primer Mix (provided with the kit) were added per reaction mix from step 1.
4. Components were thoroughly mixed and stored on ice.
5. Reverse transcription mixtures were incubated for 15 min at 42°C.
6. Tubes were put into 95°C cycle for 3 min to turn off Quanti-script Reverse Transcriptase, followed by placing all tubes on ice and storage at -20°C if needed.

3.3 Real-time polymerase chain reaction (RT-PCR)

Ready-to-use primers with the RefSeq accession numbers NM_006908 for Rac1 (catalog number PPH00733F-200), NM_001663 for Arf6 (PPH10416A-200), NM_002417 for Ki-67 (PPH01024E-200), and NM_002046 for GAPDH (PPH00150F-200) wer purchased from Qiagen. Reactions were carried out utilizing a Light Cycler (PCR System 96, Roche, Basel, Switzerland), using the Light Cycler FastStart DNA Master Plus SYBR Green provided in a kit (catalog number #03752186001, Roche). Each reaction contained 5 µL LightCycler SYBR

Green 1 (provided with the kit), 1 µL ready-to-use primer, 2.5 µL cDNA sample, and 1.5 µL RNase-free water (provided with the kit).

Denaturation was performed at 95°C for 10 min, followed by 45 cycles each including 15 sec at 95° for denaturation and 60 sec at 60° for annealing and extension. Finally, PCR product quality was demonstrated by post-PCR melt curve analysis, and samples were cooled down.

Expression values were expressed utilizing the CP (crossing point) value which indicates the number of cycles required to reach the fluorescence threshold defined by the device. ΔCP values were computed by deducting the crossing point value of GAPDH from crossing point value of the targeted gene in each sample ($\Delta CP = CP_{measured\ gene} - CP_{GAPDH}$). The values were then presented as $2^{-\Delta CP}$ and standardized to the mean values of respective controls, yielding relative $2^{-\Delta CP}$.

4 CCK8 Viability Assay

CCK8 assays for assessment of viability were performed using the Cell Counting Kit-8 (CCK-8) (Sigma Aldrich, Munich, Germany), according to the following protocoll:

1. Cell suspensions were prepared, and numbers of cells were counted. 100 µL of cell suspension containing 5,000 cells was seeded per well of 96-well plates, followed by incubation for 24 h.
2. Transfection was conducted as described above.
3. After 5 h, the old medium added with siRNA transfection mixture and without penicillin/streptomycin solution in each well was discarded and replaced by 100 µL pre-warmed medium containing penicillin/streptomycin solution.
4. At the intended time points including 24 h, 48 h and 72 h, 10 µL of CCK8 reagent (provided with the kit) was added into each well, followed by incubation for 2 h.
5. Optical densities (OD) at 450 nm in all wells were determined using a microplate reader.
6. Results are reported as ODs in diagrams, and as percentage decreases (from means for controls) in the text.

5 EDU Assay

5-ethynyl-2′-deoxyuridine (EDU) assays for the determination of proliferation rate were performed using the EDU-Click 555 Proliferation Assay Kit (catalog number BCK-EdU555IM100, Baseclick, Tutzing, Germany), according to the following protocol:

1. Cell suspensions were prepared, and 10,000 cells were added per well of 16-wells chamber slides, followed by incubation. Transfection was conducted when reaching 70% confluence after 24 h.
2. siRNA transfection was performed as described above.
3. 12 h after transfection, EDU reagents of the kit were added to stain the cells. The final concentration of EDU in each well was 10 µM.
4. Immediately prior to fixation, any remaining medium containing EDU staining reagent was withdrawn. Following this, cells were washed two times with PBS.
5. Any remaining PBS was removed, and 100 µL of Roti®-Histofix was added per well for fixation, followed by incubation for 20 min at room temperature.
6. The Histofix solution was removed, and cells were washed twice by 3% BSA solution diluted in PBS.
7. The supernatant was aspirated, and 100 µL of 0.5% Triton™ X-100 solution was added per well before incubation at room temperature for 20 min.
8. After discarding the Triton™ X-100 solution, cells were washed twice as described above. Subsequently, cells were stained by an EDU assay cocktail, using 30 µL per well for 40 min without light. The cocktail (for 12 wells) contained 100 µL reaction buffer, 40 µL catalyst solution, 2 µL dye azide and 100 µL buffer (all provided with kit).
9. The EDU cocktail solution was removed from each well, followed by washing two times with BSA solution. Subsequently, cells were stained with 30 µL (1 µg/mL) 4′,6-diamidino-2-phenylindole (DAPI) (D1306, ThermoFisher) per well in the dark at room temperature around 10 min.
10. The chambers were removed carefully with tweezers and scissors after washing each well with BSA solution. Subsequently, glass slides were gelled.

11. Fluorescence was analyzed using a laser scanning microscope (Leica LASX, Leica, Wetzlar, Germany), within two weeks after staining, with 200-fold magnification, at wavelengths of 554 nm for EDU and 405 nm for DAPI.
12. Red and blue dots seen on laser microscope images were counted using the software Image J. The proliferation rate was calculated as the proportion of red dots to the total number of dots including blue dots and red dots, multiplied by 100%.

6 Phalloidin Assay

Phalloidin staining for visualization of polymerized actin was performed according to the following protocol:

1. Cell suspensions were prepared, and 10,000 cells were added per well of 16-wells chamber slides, followed by incubation. Transfection was conducted after 24 h, when 70% confluence was reached.
2. siRNA transfection was performed as described above.
3. Immediately prior to fixation, any remaining medium was discarded, followed by washing two times with PBS per well.
4. 100 µL of Roti®-Histofix was added per well after removing PBS, followed by incubation for 20 min at room temperature to achieve fixation.
5. After removing Histofix solution, cells were washed twice by 3% BSA solution.
6. 100 µL of 0.5% Triton™ X-100 solution was added per well after removing reagents inside. Subsequently, cells were incubated at room temperature around 20 min.
7. All liquids inside were discarded, and cells were washed twice again with 3% BSA solution diluted in PBS. Subsequently, cells were stained with Fluorescein Isothiocyanate- (FITC-) labeled phalloidin (catalog number 49409, Sigma-Aldrich), using 30 µl of a 100 µM phalloidin solution per well, followed by incubation under dark conditions for around 40 min.
8. Subsequently, staining was finished by adding 30 µL DAPI without light at room temperature, lasting for 10 min after washing wells twice.

9. Chambers were carefully lifted off after discarding all liquids inside wells, and glass slides were mounted using fixing gel.
10. Fluorescence was analyzed using a laser scanning microscope (Leica LASX, Leica), within two weeks after staining, with 200-fold magnification, with 400-fold magnification, at wavelengths of 495 nm for FITC and 405 nm for DAPI. As a multicolor labeling approach was applied (Phalloidin + DAPI), it was necessary for quantification of F-actin to separate the channels and retain only the signal channel for F-actin by Image J. The detection range of actin filament signals was then set using the "Threshold" tool in Image J. The proportion of the area covered by actin filament signals to the total area in the field of view was expressed as the “phalloidin-stained area (%)”.

7 Sulforhodamine B Assay

The sulforhodamine B (SRB) method allows to assess cellular growth in cell-based studies, which may reflect the sum of proliferation, viability, apoptosis, cell death, survivial, cytotoxicity and other factors. In this study, SRB colony assay was conducted to investigate the impact of different specific siRNAs on the colony formation of hBSMCs. Colony formation assays were performed using the following protocol:

1. Cell suspensions were prepared, and 100 cells were seeded per well of the 6-well plates, filled with SMCM medium without Penicillin/Streptomycin solution for incubation.
2. After 24 h, siRNA transfection was performed as described above.
3. Medium was removed from the wells, and cells were fixed overnight by trichloroacetic acid solution (2 mL per well, 4°C), 14 days after transfection.
4. Subsequently, 6-well plates were stained by 0.4% SRB solution (2 mL per well) for 45 min at room temperature after five times washing.
5. Each well was washed five times with 1% acetic acid (2 mL per well).
6. Images were taken, and the number of colonies per well were counted.

8 Cell Contraction Assay

Cell contractions in collagen matrix plugs were evaluated using the CytoSelect™ 24-Well Cell Contraction Assay Kit (CBA-5020, Cell Biolabs, San Diego, CA, USA). The changes in the size of floating collagen reflects variations in cell contraction. The floating collagen plugs were recorded at defined time points (0.5 h, 1 h, 3 h, 6 h after seeding of cells to matrix plugs), and analyzed using ImageJ. The contraction assays were conducted according to the following protocol:

1. Cell suspension with a concentration of 1 million cells per milliliter were prepared.
2. Per well, 128.5 µL of this cell suspenstion was combined with a mix containing 397.5 µL collagen solution, 102.5 µL PBS and 14.16 µL neutralization solution per well (all provided with the kit).
3. 500 µL of this mixture were added per well of the 24-well plates (provided with the kit).
4. Plates were put back to the incubator for 1 h (37 °C).
5. After 1 h, 1 ml of SMCM medium was added to the top of each well. If experiments included agonists (carbachol, endothelin-1, or U46619) or tolterodine, these were also added along with medium in this step. Stock solutions of the agonists, including endothelin-1, carbachol, and U46619, were 1 mM, 0.1 mM, and 0.01 mM, respectively. The stock solution of tolterodine was 0.1 mM. The final concentrations of these drugs (endothelin-1, carbachol, U46619, and tolterodine) were 10 µM, 3 µM, 300 nM, and 100 nM, respectively. Thus, the volumes of drugs added per well were 10 µL, 30 µL, 30 µL, and 10 µL, respectively.
6. Cell contraction was monitored by capturing images using a camera, at 0.5 h, 1 h, 3 h and 6 h after adding SMCM. The pictures were assessed by Image J to analyze the well diameters, and the collagen diameters. Plug diameters were extrapolated to diameters in mm, based on given well diameters of 15 mm. Finally, contractions were expressed in mm, as the difference of well to plug diameter.
